# Vocal panting: a novel thermoregulatory mechanism for enhancing heat tolerance in a desert-adapted bird

**DOI:** 10.1038/s41598-020-75909-6

**Published:** 2020-11-03

**Authors:** Anaïs Pessato, Andrew E. McKechnie, Katherine L. Buchanan, Mylene M. Mariette

**Affiliations:** 1 Centre for Integrative Ecology, School of Life and Environmental Sciences, Deakin University Geelong, Geelong, VIC 3216 Australia; 2 South African Research Chair in Conservation Physiology, South African National Biodiversity Institute, Pretoria, 0001 South Africa; 3 DST-NRF Centre of Excellence at the FitzPatrick Institute, Department of Zoology and Entomology, University of Pretoria, Pretoria, 0001 South Africa

**Keywords:** Social evolution, Behavioural ecology, Climate-change ecology, Ecophysiology

## Abstract

Animals thriving in hot deserts rely on extraordinary adaptations and thermoregulatory capacities to cope with heat. Uncovering such adaptations, and how they may be favoured by selection, is essential for predicting climate change impacts. Recently, the arid-adapted zebra finch was discovered to program their offspring’s development for heat, by producing ‘heat-calls’ during incubation in hot conditions. Intriguingly, heat-calls always occur during panting; and, strikingly, avian evaporative cooling mechanisms typically involve vibrating an element of the respiratory tract, which could conceivably produce sound. Therefore, we tested whether heat-call emission results from a particular thermoregulatory mechanism increasing the parent’s heat tolerance. We repeatedly measured resting metabolic rate, evaporative water loss (EWL) and heat tolerance in adult wild-derived captive zebra finches (n = 44) at increasing air temperatures up to 44 °C. We found high within-individual repeatability in thermoregulatory patterns, with heat-calling triggered at an individual-specific stage of panting. As expected for thermoregulatory mechanisms, both silent panting and heat-calling significantly increased EWL. However, only heat-calling resulted in greater heat tolerance, demonstrating that “vocal panting” brings a thermoregulatory benefit to the emitter. Our findings therefore not only improve our understanding of the evolution of passerine thermal adaptations, but also highlight a novel evolutionary precursor for acoustic signals.

## Introduction

Hot deserts support high faunal biodiversity, in spite of extremely high air temperatures, intense solar radiation and scarce rainfall^1,2^. Organisms inhabiting these environments have evolved a suite of morphological, behavioural and physiological traits to be able to maintain a viable body temperature under extreme heat^1,3,4^. Whereas nocturnality considerably reduces heat exposure for mammals and reptiles in hot deserts, birds, being mostly diurnal, may have to tolerate operative temperatures sometimes exceeding 50 °C^5,6^. Desert birds therefore rely on highly efficient heat dissipation mechanisms capable of maintaining their body temperature up to 10–20 °C below environmental temperature for several hours each day^7^. Yet, much remains to be established on how selection may be acting upon such thermoregulatory mechanisms, and whether adaptation may partly mitigate the risk that unprecedented conditions under climate change will exceed avian physiological limits^8–11^.

The mechanisms of heat dissipation vary across avian orders and occur via three major evaporative cooling avenues: gular flutter (e.g. in nightjars), cutaneous evaporation (e.g. in doves) and panting (e.g. in passerines)^12,13^. The efficiency of panting is substantially lower than those of gular flutter or cutaneous evaporative water loss^14–16^. Indeed, the increase in respiratory frequency during panting typically involves a much higher increase in metabolic heat production (due to large thoracoabdominal muscle mass) than the rapid pulsation of the hyoid apparatus during gular flutter^17–19^. Passerines are thus an evolutionary puzzle: despite being one of the most successful avian evolutionary radiations including in arid environments^20,21^, their performance in the heat is constrained by the absence of gular flutter or the capacity for large increases in cutaneous evaporation^12,22^. The reasons why these efficient evaporative cooling pathways are absent in some avian orders, including passerines and parrots, are unclear. Interestingly however, parrots—passerines’ closest relatives^23^—have been reported to use lingual flutter (vertical movements of the tongue at frequencies synchronised with panting), as a means to augment panting efficiency^24,25^. This reveals the potential for evaporative cooling mechanisms to evolve that partly mitigate the inherent inefficiency of panting and thus augment heat tolerance. Nonetheless, to date, no such mechanism has been identified in passerines.

Recently, in a desert-adapted passerine species, adults were found to emit an unusual vocalisation, specifically triggered by high air temperatures^26–28^. This “heat-call” occurs both in wild and captive populations of the Zebra finch (*Taeniopygia guttata*), a common iconic species of the Australian arid interior^29^. Although heat-calling was first discovered during incubation—and found to adaptively program offspring for heat^26^—it is now known, in wild and captive populations, to also be produced spontaneously by heat-stressed adults, even when alone and without an active nest in non-breeding birds^27^. This begs the question as to which other function this peculiar behaviour may fulfil in the emitter, and whether it might be implicated in thermoregulation during acute heat exposure. Heat-calls are indeed very distinct from any other zebra finch vocalizations: they are high pitched, follow a very fast rhythm^26,30^ and importantly, are produced *during* panting. Even though zebra finches can also pant silently, the production of a sound during heat-calling must indicate that something in the individual’s respiratory tract changed during this specific panting bout. Similarly to gular flutter and lingual flutter, which respectively involve fast vibratory movements of the hyoid bone (in the throat) or tongue, for thermoregulatory purposes^12,24,25^, heat-calls could conceivably be produced as a side effect of vibrating an element of the respiratory tract. If so, we hypothesise that heat-calls may indicate the use of a so-far undescribed thermoregulation mechanism—or “vocal panting”—which may in turn increase individuals’ thermoregulation capacities in the heat.

Understanding the evolution and adaptive significance of thermoregulatory mechanisms requires establishing the sources of phenotypic variability in these traits. Whilst between-species variation in thermoregulatory capacities at high temperatures is well-established^31–34^, variation within species has only recently started to be widely recognised^34–37^, with phenotypic plasticity emerging as a significant driver^38^. However, whether the use and efficiency of thermoregulatory mechanisms is an individual-specific trait has, to our knowledge, never been tested. Yet, estimation of within-individual repeatability is needed to predict how selection may act on thermoregulation capacities, and potentially shape species adaptation to climate change^39^.

**Figure 1:**
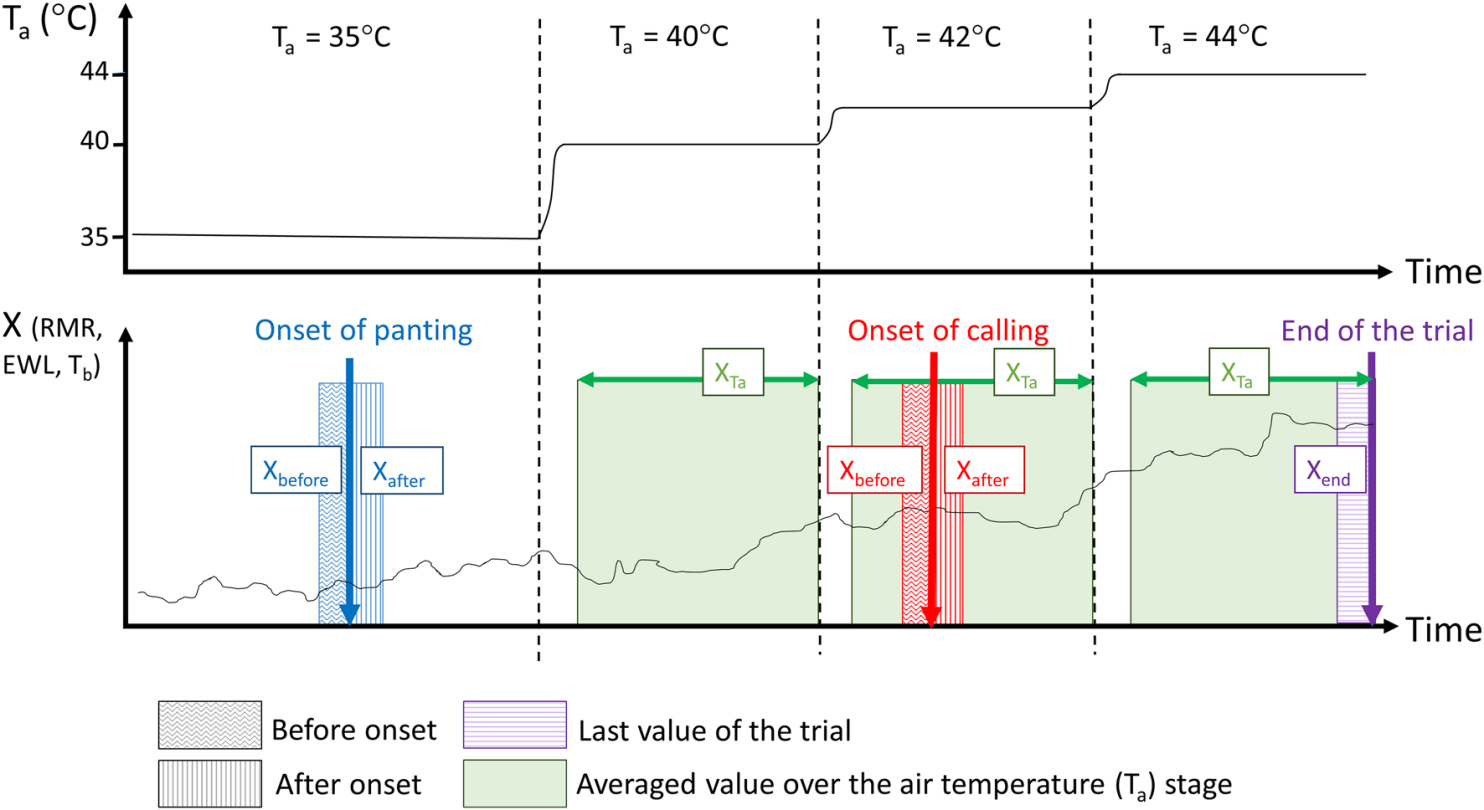
Diagram of the different respirometry values taken throughout a trial (bottom), over a stepped profile of increasing air temperature (T_a,_ top) in zebra finches tested in a flow-through respirometry system with high flow rates. Instantaneously corrected values at the onset of panting (3-min windows before and after, in blue), at the onset of calling (3-min windows before and after, in red), and averaged values over stable air temperature stages (~ 14 min, in green) were used. We also calculated values at the end of the trial (last 3-min window, in purple) to verify *a-posteriori* the validity of our trial interruption criteria. See supplementary material for details.

Here, we tested whether vocal panting may represent a so-far unknown thermoregulatory mechanism in desert-adapted passerines, and whether individuals vary in the degree to which they rely on such a thermoregulation strategy. To this aim, we repeatedly exposed 44 male and female adult wild-derived captive zebra finches to increasing air temperature (T_a_) in an open flow-through respiratory system, in which humidity levels were maintained suitably low (water vapour partial pressure < 0.72 kPa in excurrent air)^32^. During each trial, T_a_ increased gradually up to 44 °C (i.e. just below potentially lethal temperatures at 45–46 °C^40^) following a stepped profile of consecutive ‘T_a_ stages’ at 27 °C, 35 °C, 40 °C, 42 °C and 44 °C (Fig. 1). Similarly to recent studies on avian thermoregulation during acute heat exposure^14,15,37^, we measured the resting metabolic rate (RMR), evaporative water loss (EWL), and body temperature (T_b_) of individuals as they started to pant or emit heat-calls, and throughout the temperature gradient (Fig. 1). We predicted that if heat-calling is associated with a particular thermoregulatory mechanism, (1) heat-calling will occur after the onset of silent panting, at predictable (i.e. repeatable) air or body temperatures for any given individual, (2) heat-calling onset will coincide with an increase in EWL (as also observed at the onset of panting^37^), and possibly with changes in RMR and (3) ultimately, heat-calling individuals will show higher heat-tolerance (defined as being more likely to reach and tolerate the highest air temperature, without showing severe signs of heat-stress prompting the interruption of the trial), although such heat-tolerance benefit may come at a cost of greater total water loss.

## Results

### Triggers and repeatability of panting and heat-calling

**Figure 2:**
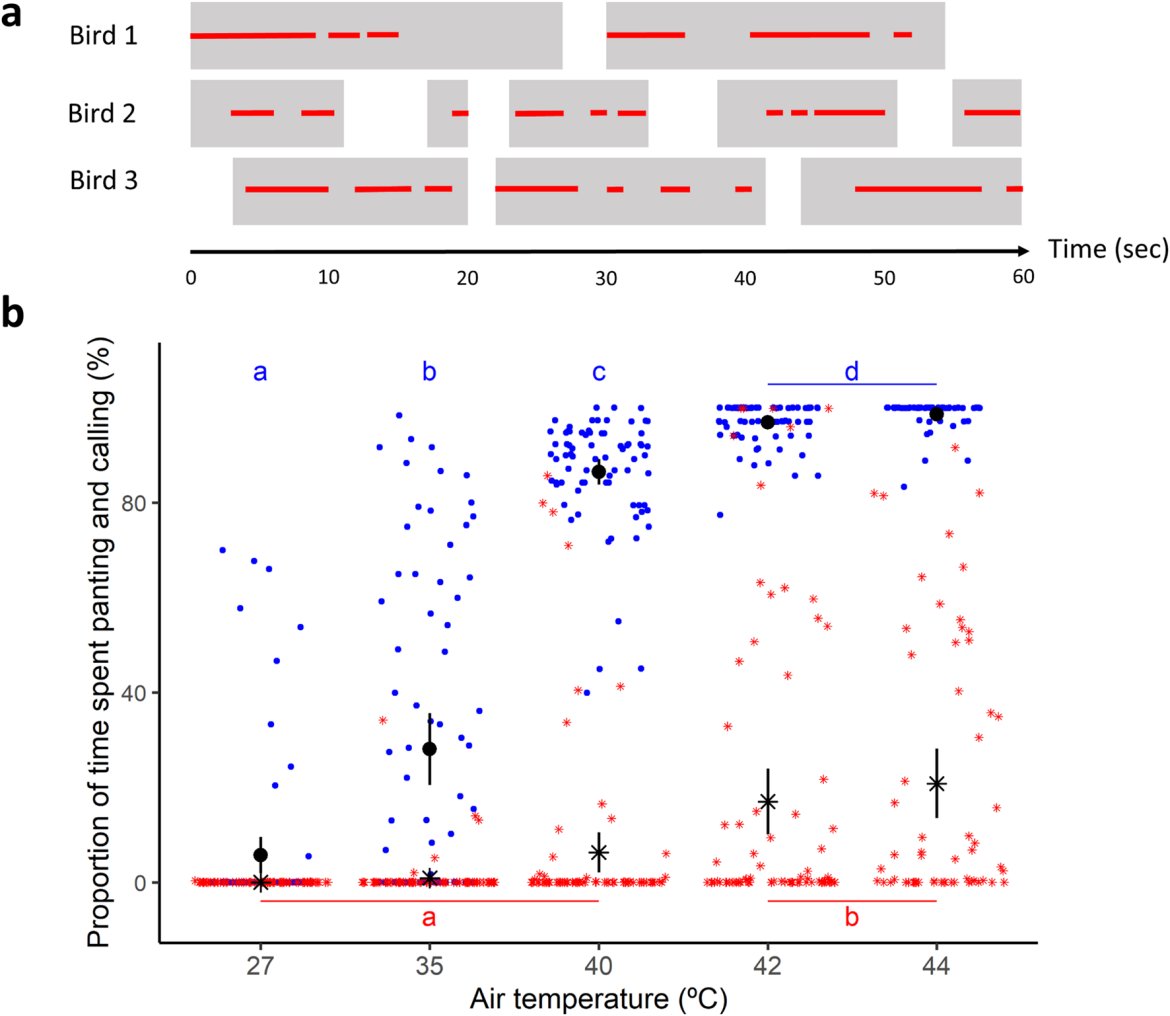
Panting and heat-calling occurences in wild-derived captive zebra finches. (**a**) Examples of 1-min windows of three birds (row) in which the individual pants on and off (grey filling) and also calls on and off (red lines) within each panting bout. (**b**) Proportion of time spent panting (blue dots) and calling (red asterisks) by individual zebra finches exposed to a stepped profile of increasing air temperature. The mean calling (black asterisk) or panting (blue dot) times ± SE are shown (n = 77 trials for 44 birds). Letters indicate significant differences of time spent panting (blue) and calling (red) between each T_a_ stage (p < 0.05) according to post-hoc tests.

Heat-calls were emitted during at least one trial by 73% (i.e. 32 out of 44) of the birds. Birds were always panting when they started heat-calling and produced heat-calls within panting bouts as they continued to pant (Fig. 2A). On average, heat-calling started 39.93 ± 3.23 min after silent panting. Heat-calling onset thus occurred at higher T_a_ and T_b_ (T_a_ = 40.84 ± 0.38 °C, range 27–44 °C; and T_b_ = 43.20 ± 0.12 °C (range 41.36–45.14 °C) than silent panting (T_a_ = 36.23 ± 0.49 °C, range 27–40 °C; and T_b_ = 41.74 ± 0.09 °C, range 39.98–43.05 °C). Overall, the proportion of time each individual spent panting increased gradually across T_a_ stages until reaching its maximum at T_a_ stage = 42 °C (LMM, p < 0.001, Fig. 2B), whereas the proportion of time each individual spent calling remained low and then increased sharply at T_a_ = 42 °C (LMM, p < 0.001, Fig. 2B).

**Figure 3:**
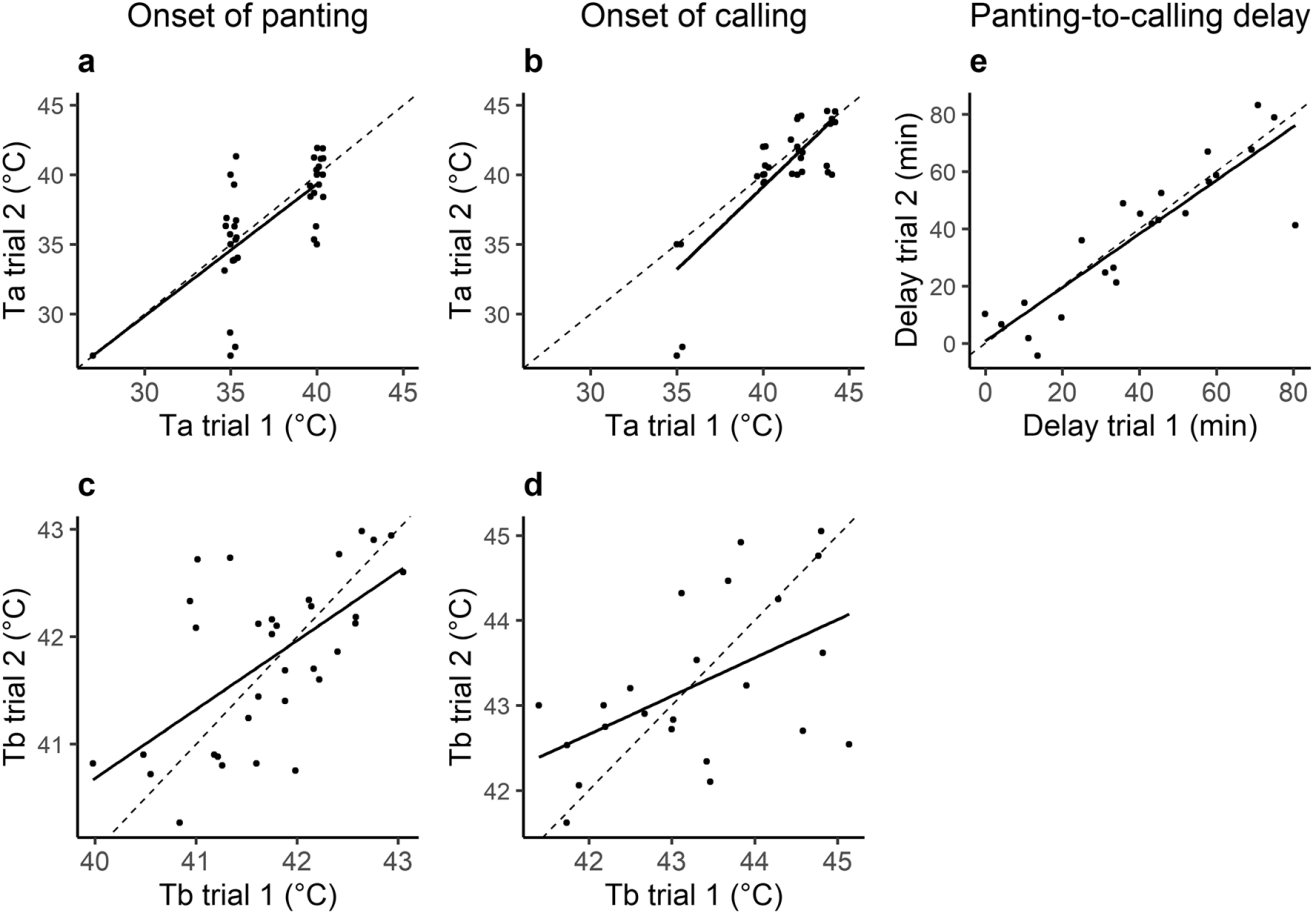
Within individual repeatability of (**a**,**b**) air temperature (T_a_) and (**c**,**d**) body temperature (T_b_) thresholds triggering the onset of silent panting (**a**,**c**, n = 33 birds) or heat-calling (**b**,**d**, n = 23 birds), and (**e**) panting-to-calling delay (n = 23 birds). For T_a_ (**a**,**b**) points are slightly jittered for clarity. Plain lines correspond to regression line estimated with lm method in *ggplot2* and dashed lines correspond to R^2^ = 1.

Of the calling birds tested twice, 88% (i.e. 23 out of 26) called during both trials. The T_a_ and T_b_ thresholds for the onset of silent panting and heat-calling were highly repeatable within individuals (respectively for T_a_ and T_b_, silent panting: ρ = 0.80, p < 0.001 and cor = 0.61, p < 0.001, n = 33 birds; heat-calling: ρ = 0.69, p < 0.0001 and cor = 0.521, p = 0.011, n = 23 birds, Fig. 3A–D), even though trials were conducted two weeks apart. Interestingly, the time elapsed between the onsets of panting and calling (i.e. panting-to-calling delay) was also remarkably repeatable within individuals (cor = 0.88, p < 0.001, n = 23 birds; Fig. 3E). Together these results show that although individuals varied in their reliance on heat-calling, they resorted to this strategy at a precise, individual-specific, stage of the heat challenge.

### Thermoregulatory changes associated with panting and heat-calling

We examined the changes associated with silent panting and heat-calling over two time scales. Over a short time scale, we compared thermoregulatory variables during the 3 min preceding and the 3 min following the onset of panting or calling (see methods, Fig. 1). Over a longer time scale, we measured the effects of the proportion of time spent panting or heat-calling on thermoregulatory variables during entire stable T_a_ stages (i.e. ~ 14 min, see methods, Fig. 1).

**Figure 4:**
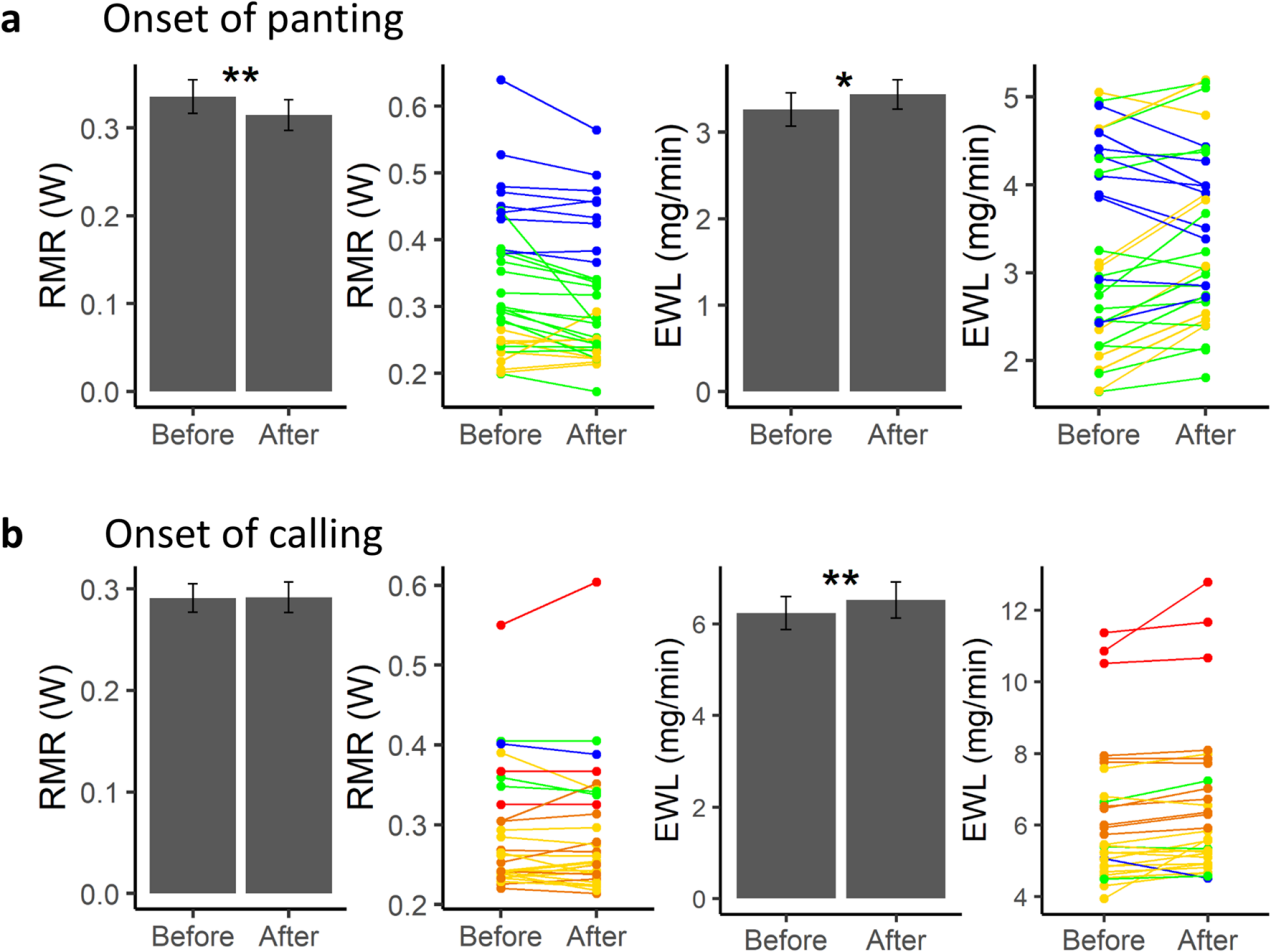
RMR and EWL changes in the 3-min window at the onset of (**a**) silent panting (n = 32 trials for 24 birds) and (**b**) heat-calling (n = 29 trials for 23 birds). Colours correspond to air temperatures (T_a_): blue = 27 °C, green = 35 °C, yellow = 40 °C, orange = 42 °C, red = 44 °C. Error bars represent mean ± SE (*p < 0.05, **p < 0.01).

For silent panting, EWL increased both in the 3 min following the onset of panting (p = 0.027, n = 32 trials for 24 birds, Fig. 4A, Supplementary Table 1) and with panting duration over T_a_ stages (p = 0.001, Table 1). However, effects on RMR were variable: RMR significantly decreased just after the onset of panting (p = 0.004, Fig. 4A, Supplementary Table 1) but did not change over T_a_ stages (p = 0.237, Table 1).

**Table 1:** Effects of the proportion of time spent panting or calling at each air temperature (T_a_) stage above the thermoneutral zone on the mean thermoregulatory values during the corresponding T_a_ stage (c.a. 14 min each; n = 140, 44 birds).

Predictors	RMR_Ta_			EWL_Ta_		
	est	SE	p	est	SE	p
**Panting**						
Intercept	0.20	0.01	** < 0.001**	2.78	0.24	** < 0.001**
Proportion panting	0.00	0.00	0.237	0.25	0.07	**0.001**
Mass	0.01	0.00	** < 0.001**	0.24	0.11	**0.038**
Sex	0.00	0.01	0.668	0.22	0.24	0.357
T_a-std_	0.04	0.00	** < 0.001**	2.29	0.10	** < 0.001**
**Calling**						
Intercept	0.28	0.01	** < 0.001**	7.33	0.17	** < 0.001**
Proportion calling	0.01	0.00	**0.003**	0.18	0.07	**0.017**
Mass	0.02	0.00	** < 0.001**	0.28	0.11	**0.012**
Sex	0.00	0.01	0.811	0.21	0.23	0.363
T_a-std_	0.04	0.00	** < 0.001**	2.39	0.09	** < 0.001**

Bold values indicate significant effects (p < 0.05).

During heat-calling, EWL increased at both timescales (3-min timescale: p = 0.005, n = 29 trials for 23 birds, Fig. 4B, Supplementary Table 1; T_a_ stage timescale (14 min): p = 0.017, Table 1). RMR also increased with the proportion of time spent calling over a T_a_ stage (p < 0.003, Table 1) but no increase in RMR was detected right at the onset of calling (p = 0.829, Fig. 4B, Supplementary Table 1).

Overall, both heat-calling and panting were associated with increases in EWL. In contrast, changes in RMR differed between calling and panting, likely because most individuals had not reached their lowest RMR values by the time they started silent panting (at T_a_ = 27 °C and 35 °C), whereas calling onset (at T_a_ = 40 °C and above) occurred after RMR had stabilised. Consistent with this interpretation, we found that when restricting analyses to trials where individuals started silent panting at T_a_ = 40 °C (n = 8 trials for 6 birds), RMR did not change at the onset of panting (LMM: est < 0.01, se = 0.01, p = 0.704).

### Effect of panting or heat-calling on heat tolerance and water loss

Birds in 75% of the trials reached T_a_ = 44 °C, whereas other trials had to be interrupted at T_a_ = 42 °C because the bird showed signs of severe heat stress (e.g. loss of balance or rapid increase of T_b_). Among trials reaching T_a_ = 44 °C, only 27% were completed, with the individual tolerating T_a_ = 44 °C for 20 min. Importantly, the likelihood of completing a trial was highly repeatable within individuals (R = 0.97, p < 0.001, n = 33 birds). Furthermore, the birds completing the trial had significantly lower RMR_end_, EWL_end_ and T_b-end_ values in the last 3 min of their trial than those that had to be interrupted, confirming the validity of our interruption criteria (see methods).

**Figure 5:**
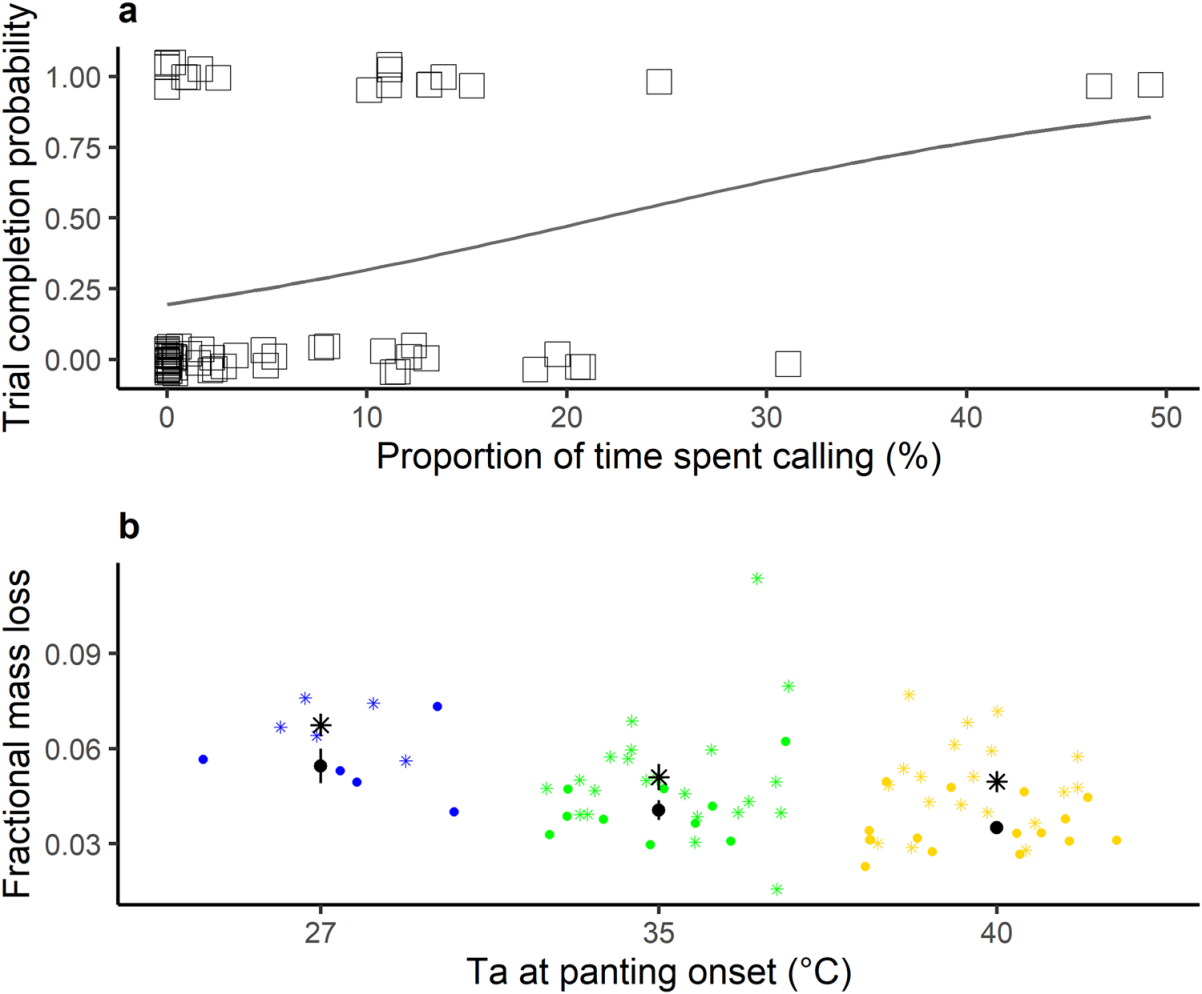
Thermal benefits and costs of panting and heat-calling. (**a**) Effect of the proportion of time spent calling on the individual’s ability to complete the trial (0 = not competed, 1 = completed; n = 77 trials for 44 birds). Full line corresponds to the change in probability to complete the trial estimated with glm (generalized linear model) method in *ggplot2*. (**b**) Fractional mass loss in relation to the air temperatures (T_a_) at which individuals started panting (blue = 27 °C, green = 35 °C and yellow = 40 °C) and whether or not they emitted heat-calls (dots = non-calling individuals, asterisks = calling individuals, n = 77 trials for 44 birds). Points are jittered for clarity. Black dots/asterisks and error bars represent mean ± SE.

Critically, in support of our prediction, trial outcome was significantly related to the proportion of time spent calling: birds completing trials had called for longer (GLMM: est = 3.67, se = 1.69, p = 0.030, n = 77 trials for 44 birds, Fig. 5A). By contrast, the proportion of time spent panting had no effect on trial completion (GLMM: est = 1.28, se = 1.72, p = 0.458).

Nonetheless, the thermoregulatory benefit of vocal panting came at a cost of greater water loss, as indicated by individuals’ fractional mass loss during the trial in relation to the cumulative effects of panting and calling. Indeed, calling birds had significantly higher mass loss than non-calling birds (LMM; est = 0.16, se = 0.07, p = 0.023, n = 77 trials for 44 birds, Supplementary table 2, Fig. 5B), after accounting for the significant effect of the T_a_ at which they had started to pant (LMM; est = − 0.11, se = 0.04, p = 0.003, Supplementary table 2, Fig. 5B).

## Discussion

As predicted, we found that heat-calling (i) occurred at a predictable stage of panting, (ii) triggered changes in EWL, and (iii) importantly, was associated with a higher likelihood of tolerating acute heat exposure in adult wild-derived captive zebra finches. Our data therefore reveal a novel mechanism for thermoregulation that increased individual’s heat tolerance, supporting the hypothesis that vocal panting may offset the inherent inefficiency of panting, as an evaporative cooling pathway in a small arid-zone passerine. Furthermore, we provide the first evidence for consistent individual differences in thermoregulatory strategies, with heat-call production, as well as silent panting, being highly repeatable traits within individuals. Overall, our study makes a significant step towards a greater understanding of the selection forces that may act on thermoregulatory capacities in a desert avian species. Such advances are particularly crucial at a time when desert avian communities are highly threatened by unmitigated climate warming^8–11^. Furthermore, our findings highlight that respiratory movements for thermoregulatory purposes may represent a novel evolutionary precursor of acoustic signals.

### Vocal panting as a strategy to improve heat tolerance

Individuals showed notable differences in heat tolerance, where some individuals showed signs of distress at T_a_ = 42 °C, whereas others tolerated T_a_ = 44 °C for up to 20 min. Importantly, individuals’ capacities to tolerate the highest T_a_ and complete the trial increased with the proportion of time they spent heat-calling (Fig. 5A), whereas panting duration had no such effect. Nonetheless, EWL increased at the onset of vocal panting compared to silent panting, and calling birds lost more mass than non-calling birds, indicative of elevated water loss throughout the trial (Fig. 5B). Taken together, our findings therefore demonstrate that vocal panting—or the thermoregulatory mechanism associated with heat-calling—improves heat tolerance by increasing evaporative water loss. Interestingly, the onset of heat-calling in our experiment at T_b_ ~ 43 °C coincides with the T_b_ at which zebra finches have classically been described to reach hyperthermia^41^. At moderately high air temperatures, avian T_b_ increases to maximise passive heat loss through non-evaporative avenues and thus save water^31,42^. A previous classic study postulated that, above T_b_ = 43 °C nonetheless, zebra finches would favour water loss over detrimental effects of hyperthermia^41^. Our results suggest that vocal panting may correspond to such change in thermoregulation strategy predicted by that study^41^, to increase evaporative water loss when individuals are no longer able to maintain a viable T_b_ via silent panting.

### Repeatability in thermoregulation mechanisms within individuals

To the best of our knowledge, our study is the first to show the repeatability of thermoregulatory mechanisms and heat tolerance within individuals. We showed that individual heat tolerance, as measured by their capacity to reach and withstand the air temperature stage of T_a_ = 44 °C, was remarkably repeatable between two trials conducted 2 weeks apart. In addition, in agreement with findings on basal metabolic rate^43,44^, our analyses reveal that T_a_ and T_b_ thresholds triggering panting, as well as heat-calling, were highly consistent within individuals. Furthermore, we showed each individual triggered heat-calling at a specific instant after silent panting had started, with the panting-to-calling delay varying up to eightfold between individuals but being extremely repeatable within individuals. This further suggests that heat-calling is tightly linked to thermoregulation, and specific to the individual. These findings also indicate that, under controlled conditions, individuals consistently differ in how they use thermoregulatory mechanisms to withstand a heat challenge. This points to the occurrence of individual-specific thermoregulatory strategies within populations, even though individual thermoregulatory capacities may vary plastically across seasons or acclimation conditions^38,45,46^. Importantly, to limit sources of inter-individual variation at adulthood, all individuals tested in this study were of similar age (4 year-old) and thermal experience (always held together in the same outdoor aviaries). However, it is currently not clear whether the inter-individual differences we found in adult thermoregulation strategies and the production of heat-calls were due to differences in genotypes, or induced by developmental plasticity in early life. In particular, future studies may establish whether prenatal exposure to heat-calls and/or high temperatures during development favours the production of heat-calls at adulthood or improves individuals’ thermoregulation capacities. Nonetheless, our findings that thermoregulatory behaviour consistently vary between individuals open the possibility for selection to act on this trait^47,48^.

### The evolution of vocal panting

Our findings reveal the existence of a novel modification of panting in a passerine species, which improves thermotolerance. It is unlikely that vocal panting is a phenomenon that uniquely evolved in zebra finches. For example, it is noteworthy that other Australian passerines such as fairy wren species, breeding in hot climates, produce similar fast, high-pitch calls during incubation, even though their possible association with heat (or thermoregulation) has not been investigated^49,50^. We predict that vocal panting may also occur in other small-sized heat-adapted passerines, where the reliance on facultative hyperthermia is common^31^. Furthermore, given that vocal panting is costly in terms of water loss, it may be more likely to occur in drinking species than non-drinking species^51,52^. Therefore, examining the occurrence of heat-calling across passerines species occupying hot environments may bring new insights on the resilience of desert avian communities to climate warming.

In addition, our study provides evidence that a vocal signal can be associated with a thermoregulatory mechanism. Whilst this may seem surprising, there is evidence for a similar phenomenon occurring with visual signals. Indeed, some visual cues, such as feather erection or skin flushing (i.e. redness of highly vascularised skin patch), that were primarily associated with a thermoregulation function, have then evolved to become visual signals during social displays in some species^53–55^. In our study, consistent with previous findings in wild and captive zebra finches^26,27^, we showed that heat-calls, starting at T_a_ ~ 41 °C and T_b_ ~ 43 °C, can reliably signal hot environmental conditions and the emitter’s hyperthermic state to embryos (or possibly to conspecifics). Therefore, it is plausible that heat-calls could have evolved from a cue initially associated with a thermoregulatory mechanism into a signal directed at embryos, which increases offspring fitness. Such a hypothesis is consistent with the evidence showing that heat-calling occurs in multiple contexts but is most common towards the end of incubation, when embryos might be able to perceive that signal^26,28^.

Nonetheless, the mechanisms for heat-call production and its associated evaporative cooling benefits remain to be established. For example, contrary to anecdotal evidence on a single individual zebra finch suggesting that respiration rate might increase during heat-call production^28,56^, we found no supporting evidence for a rapid increase in RMR at calling onset. Moreover, the mode of heat-call production requires further investigation. For example, a possible mechanism underlying the increase in EWL and thus thermoregulatory benefits of vocal panting is that, similarly to gular flutter during panting^19^, heat-calling increases the area for evaporative water loss by moving air across parts of the respiratory tract that are not usually involved in panting. However, we currently do not know where the sound originates in the respiratory tract and which element might be vibrating, although the involvement of the medial and lateral labia, which are known to vibrate to produce sound^57^, are worth investigating. Overall, establishing the mechanical pathway for sound production during heat-calling may give insights into its associated thermoregulatory benefits.

## Conclusion

Our study reveals the intriguing phenomenon of vocal panting, whereby a vocalisation can be associated with a thermoregulatory mechanism which improves heat tolerance. This, together with the adaptive developmental effects of heat-call exposure on zebra finch embryos^26^, add to the remarkable adaptations displayed by species exposed to extreme environmental conditions. Whilst more investigation is needed to understand the underlying mechanisms of vocal panting, as well as heat-call occurrence among avian taxa, our findings suggest that, beside panting, some passerine species are likely to show additional thermoregulatory mechanisms for heat tolerance, perhaps analogous to lingual flutter in parrots^24,25^. Overall, our study highlights the importance of understanding how thermoregulatory mechanisms vary within species, in order to predict selection on heat tolerance and how species may fare under climate change. Indeed, future climate projections have shown that the increase in chronic hot weather will threaten the persistence of bird populations inhabiting arid environments, by dramatically increasing breeding failure^10^. In addition, the risk of lethal dehydration and hyperthermia is predicted to increase dramatically in these environments for small passerines species, including the zebra finch^8,9^. Therefore, as our study hints to how much remains to be discovered, understanding avian thermoregulatory mechanisms and their implications for adaption to hot climates is more important than ever.

## Materials and methods

### Study species and housing

Between March and June 2018, 44 adult wild-derived zebra finches (26 males and 18 females) were transferred from outdoor aviaries to indoor cages (4–5 same-sex individuals per cage; 1 × 0.42 × 0.42 m) at Deakin University (Geelong, Australia) for acclimation to a constant temperature of 25 °C and photoperiod of 12L:12D. Birds were supplied with ad libitum finch seed mix, grit, cucumber and water. After three days, a temperature-sensitive passive integrated transponder (PIT) tag (Biomark, Boise, USA) was inserted subcutaneously onto the bird’s flank, which have been demonstrated to give similar T_b_ to those implanted intra-peritoneally in zebra finches^58^. Physiological measurements took place after 31 days (range 27–36 days) of acclimation for the first trial (with the exception of a female after 96 days) and after 47 days (range 42–51 days) of acclimation for the second trial, with an average of 16 days (range 6–24 days) between the two trials.

### Experimental protocol

Experimental trials were conducted during the day, when the birds would naturally experience higher temperatures. Thirty-three birds were tested twice, once in the morning (start 10:30 am ± 13 min) and once in the afternoon (start 2:50 pm ± 16 min) in random order. An additional 11 birds were tested only once due to time constraints. To maximise the likelihood of birds being post-absorptive during measurements, birds were fasted for two hours before the trial^59^ with ad libitum water in an individual cage (32 × 50 × 33 cm), in auditory and visual contact with others in the holding room. Birds were then weighed (g ± 0.01, Sartorini scale) before being placed in the metabolic chamber. During measurements, air temperature was initially maintained at 27 °C for 25 min (or 45 min for the second trial), then 35 °C for 30 min (i.e. thermoneutral temperature^41^), followed by 20 min stages at 40 °C, 42 °C and 44 °C. The 27 °C stage for the second trial was longer to ensure that patterns of panting or calling were directly linked to temperature, rather than time spent in the chamber. After 10 min at T_a_ = 27 °C, we started a playback of birds in the holding room to lower stress due to social isolation as commonly done in behavioural studies^60,61^. We used an open flow-through respirometry system to measure individual CO_2_ production and EWL as previously described^32^ (see supplementary material for details).

Trials were considered ‘complete’ when the individual remained in the chamber for 20 min at T_a_ = 44 °C. The trial was stopped before this if the bird showed a persistent escape behaviour (attempting to fly, pecking container), a loss of balance, an abrupt drop in the metabolic traces or a high T_b_ (> 45 °C), as^32^. The experimenter was blind to heat-call production or to results from the previous trial. To independently confirm the validity of our interruption criteria, and that birds completing the trial were less severely heat-stressed than those in interrupted trials, we tested for differences in thermoregulatory values at the end of the trial (last 3-min, Fig. 1) between birds completing the trial and those that did not, using Linear Mixed Models (LMM) (one per thermoregulatory variable) with individual as a random factor, and trial completion (yes/no), mass and sex as fixed effects. Because some birds did not reach T_a_ = 44 °C, we had n = 7 observations at T_a_ = 42 °C and n = 25 observations at T_a_ = 44 °C for final values. Therefore, we run these analyses using data for all birds (n = 32 trials for 24 birds) or only for birds that reached T_a_ = 44 °C (n = 25 trials for 18 birds). In agreement with our decision criteria, birds in completed trials had lower thermoregulatory end values than those that had to be interrupted. Specifically, completing birds had a significantly lower RMR_end_, EWL_end_ and T_b-end_ than birds interrupted at T_a_ = 44 °C (respectively, p < 0.001, p = 0.007, p = 0.031, n = 25), and a significantly lower RMR_end_ (LMM: est = − 0.10, se = 0.03, p = 0.005, n = 32) and marginally lower T_b-end_ (LMM: est = − 0.47, se = 0.25, p − 0.067, n = 32) than birds interrupted at either T_a_ = 42 °C or 44 °C.

After the trial, birds were weighed and given water (by depositing drops of water on their bill). All birds were subsequently monitored to ensure they recovered from the experiment and had access to ad libitum water and food in their home cage.

All procedures were approved by Deakin University Animal Ethics Committee (G06-2017). All experiments were performed in accordance with Australian guidelines and regulations for the use of animals in research.

### Behavioural monitoring

Behavioural monitoring consisted of a 5-s scan every 30 s using two infrared video cameras (mini CCD camera with IR, Signet) placed into the cabinet. We scored the activity (0 = resting, 1 = looking around, 2 = moving, 3 = hopping, 4 = agitated, 5 = very agitated), as well as panting behaviour (i.e. opening of the bill^62^). We considered as the onset of panting when the individual was panting for at least three consecutives scans. Proportion of time spent panting was computed over each air temperature stage and for the total trial length.

### Acoustic recording

Heat-call production was recorded using a microphone (Seinnheiser MKE 2P, Germany) inserted through the metabolic chamber lid and connected to an external recorder (H6 handy recorder, Zoom). Audio recordings were analysed visually and audibly in Adobe Audition CC (Creative Cloud 2018) by two researchers who were blind to the respirometry values, behavioural data and air temperatures. The onset of calling corresponded to the time when the bird initiated the first heat-call sequence; and the duration of each sequence was noted as previously described^27^. We calculated the proportion of time spent vocal panting over each T_a_ stage and for the total trial duration. We also defined “calling” as a categorical variable with 1 corresponding to birds calling for a total duration of at least 20 s and 0 for birds not calling, or calling for less than 20 s. This threshold corresponded to a gap in the histogram of calling duration and allowed us to consider ambiguous individuals calling for less than 20 s as non-calling birds.

### Respirometry data processing

We calculated whole animal RMR, EWL using equations previously described^63^. Our birds being post-absorptive, we assumed a respiratory exchange ratio of 0.71^32,64^. We computed thermoregulatory values for each individual at three time points: at the onset of panting, at the onset of calling and at the end of the trial (Fig. 1, see supplementary material for details). We also computed the mean values for each T_a_ stage above the thermoneutral zone of the zebra finch (T_a_ ≥ 40 °C, Fig. 1, see supplementary material for details).

### Statistical analyses

All the analyses were performed in R (v3.5.3) on RStudio (v1.1.463). Values are shown as mean ± standard error. The total data set was 77 trials for 44 birds, including 33 birds tested twice. Nonetheless, we excluded values when individuals were agitated (activity > 3) for the analyses with thermoregulatory variables (RMR and EWL), as previously described^32^, and sample sizes were smaller in some analyses since not all birds called (see “Results”). For every model, predictors were normalised and residuals were checked for normality and homoscedasticity. Results from the full models are shown.

#### Triggers and repeatability of panting and heat-calling

To understand in which conditions heat-calling occurs, we examined the effect of T_a_ on the proportion of time spent calling and panting at each air temperature stage using separate LMMs (*lme* function from the *nlme* R package) with individual ID and trial number as random effects and T_a_ as a fixed effect (n = 370 observations for 77 trials and 44 birds), followed by post-hoc pairwise Turkey comparisons test (*emmeans* function from the *emmeans* R package)..

To investigate the link between panting and calling, we calculated the time delay between the onset of each behaviour (panting-to-calling delay). Within-individual repeatability in panting-to-calling delay between the two trials was assessed using a Pearson correlation test (n = 23 birds). In addition, within-individual repeatability in T_a_ and T_b_ thresholds triggering panting (n = 33 birds) and calling (n = 23 birds) between the two trials was assessed using either a Pearson or Spearman correlation test, depending on whether the data were normally distributed.

#### Thermoregulatory changes associated with panting and heat-calling

We first compared the instantaneous changes in thermoregulatory variables at the onset of panting and calling in separate models. We investigated the effect of the sampling window (3-min before/after the onset, Fig. 1) on RMR and EWL using LMMs with trial number nested in individual ID as a random factor, and mass, sex and T_a-std_ as fixed effects (*lme* function from the *nlme* R package). T_a-std_ corresponds to T_a_ centred on the median T_a_ stage for the focal behaviour (i.e. coded as 0 at T_a_ = 35 °C for panting and T_a_ = 42 °C for calling), with increasing or decreasing temperatures equally spaced (one increment) from the centred temperature (e.g. for calling T_a_ = 35 °C coded − 2, T_a_ = 40 °C coded − 1, T_a_ = 44 °C coded 1). Because we could not obtain values when the behaviour onset occurred during baseline measurements, the sample sizes were n = 32 trials (for 24birds) for panting onset and n = 29 trials (for 23 birds) for calling onset.

Then, to corroborate these short-term (i.e. 3-min) changes at panting and calling onsets, and to circumvent the issue of onsets occurring during baseline measurements, we assessed the effect of the proportion of time spent calling and panting per T_a_ stages on the average RMR_Ta_ and EWL_Ta_ values during these T_a_ (ca. 14 min, see above , Fig. 1), for T_a_ above the thermoneutral zone. We fitted LMMs with the proportion of time spent panting or calling as predictors in separate models, individual ID and trial number as random effects and sex, mass and T_a-std_ as fixed effects using *lme* function (n = 140 observations for 77 trials and 44 birds).

#### Effect of panting or heat-calling on heat tolerance and water loss

To determine whether panting or heat-calling improve heat tolerance (i.e. the ability to withstand high T_a_ without showing severe signs of heat-stress such as loss of balance or rapid increase of T_b_), we evaluated potential predictors of successful trial completion (i.e. remained calm with T_b_ < 46 °C for 20 min at T_a_ = 44 °C). Within-individual repeatability of trial completion probability was assessed using Generalized Linear Mixed Models (GLMMs) based repeatability estimation for binary data using *rptR* R package^65^. To test for the effect of the proportion of time spent panting or calling on the trial completion we fitted separate GLMMs with a binomial error distribution and individual as a random factor (n = 77 trials for 44 birds), and mass, sex and trial number as fixed effects.

To investigate the potential costs of panting and heat-calling on total water loss, we tested, in the same LMM model, the effects of the air temperature stage at which bird started panting (T_a-panting_) and calling occurrence (0/1) on the fractional mass loss (n = 77 trials for 44 birds). We used calling occurrence rather than the proportion of time spent calling, to allow retaining non-calling birds in the analysis. We also included mass, sex and trial completion (yes/no) as fixed effects, and individual as a random factor (*lmer* function).

## Supplementary information

Supplementary Information

## Data Availability

The datasets generated and analysed during the current study are available in Mendeley (https://dx.doi.org/10.17632/hrzdn7yjwm.1).
