# Supplementary material for: Vocal panting: a novel thermoregulatory mechanism for enhancing heat tolerance in a desert-adapted bird
Source: Sci Rep. 2020 Nov 3;10:18914. doi: 10.1038/s41598-020-75909-6 (PMC7609653; doi:10.1038/s41598-020-75909-6)
Supplement: Supplementary file 1 — Supplementary Information [file 41598_2020_75909_MOESM1_ESM.docx]

**Vocal panting: a novel thermoregulatory mechanism for enhancing heat tolerance in a desert-adapted bird**

Anaïs Pessato^1*^, Andrew E. McKechnie^2, 3^, Katherine L. Buchanan^1^, Mylene M. Mariette^1*^

^1^Centre for Integrative Ecology, School of Life & Environmental Sciences, Deakin University, Geelong 3216, Australia.

^2^South African Research Chair in Conservation Physiology, South African National Biodiversity Institute, Pretoria 0001, South Africa

^3^DST-NRF Centre of Excellence at the FitzPatrick Institute, Department of Zoology and Entomology, University of Pretoria, Pretoria 0001, South Africa

**Supplementary information**

**Materials & methods**

Gas exchange and body temperature measurements

We used an open flow-through respirometry system to measure individual CO_2_ production and EWL. The metabolic chamber consisted of a transparent plastic chamber (1.5 L, clip container, Home&CO, 8 x 18 x 11.5 cm) designed by Whitfield et al. (2015). The chamber contained a thermocouple (type K, RS PRO, Australia) to measure the air temperature, a plastic perch and a plastic mesh on top of 5mm layer of mineral oil to trap excreta. The chamber was placed into a temperature-controlled cabinet (0.96 x 1.44 x 0.60 m) regulated by a controller (ir33, Carel) connected to a thermocouple (type K, RS PRO, Australia).

We used the same respirometry setup as Whitfield et al. (2015). Briefly, a pump (Gast DAA-V515-ED, Cole-Parmer) pushed dry air into the metabolic chamber. A low humidity level in the chamber (range: 0.08-0.72 kPa in excurrent air) was maintained by regulating the airflow (range: 1-3.5 SPLM) with a mass flow controller (Alicat scientific Inc., Tuscon, USA). Baseline air was subsampled between each air temperature stage for five minutes. Baseline and air from the chamber were subsampled from a PVC tube and pulled by a pump (SS4 subsampler, Sable Systems) at 213 mL/min through a H_2_O analyser (RH-300, Sable Systems) and a CO_2_ analyser (CA-10, Sable Systems). The water analyser was calibrated every day using the O_2_ dilution technique ^62^ and the CO_2_ analyser every second day using certified gas with a known CO_2_ concentration of 2080 ppm (BOC, Australia). Water and CO_2_ analysers were zeroed with a highly pure nitrogen (Supagas, VIC, Australia). Analysers and thermistor probes were connected to an analog-digital converter (UI2, Sable Systems) to enable recording using Expedata sofware (Sable Systems). Body temperature was continuously monitored and recorded using a PIT tag reader (HPR plus, Biomark).

**Respirometry data processing (further details)**

To investigate the changes at the onset of panting and calling, we averaged RMR and EWL over three minute windows before and after the onset (blue and red in Fig. 1). We applied the instantaneous transformation to correct the gas concentrations for differences in response time ^58^. The correction values were 0.26 and 0.15 for CO_2_ and H_2_O respectively.

For each air temperature stage (as represented by ‘_Ta_’ in Fig. 1), we averaged the thermoregulatory values over the entire stage after reaching stable temperature over 15.19±0.14min at T_a_=40°C, 13.00±0.33min at T_a_=42°C and 12.26±1.01min at T_a_=44°C (RMR_Ta_ and EWL_Ta_, green in Fig. 1). Average durations at T_a_=42°C and 44°C were lower and varied because some trials were interrupted when the bird showed severe signs of heat-stress before the end of 20 minutes at these T_a_.

Lastly, to validate our interruption criteria we calculated RMR_end_, EWL_end_ and T_b-end_ by averaging the values from the last three minutes before the trial was interrupted or ended (see above, purple in Fig. 1).

**Supplementary tables**

|  | **RMR** | | | **EWL** | | |
| --- | --- | --- | --- | --- | --- | --- |
| *Predictors* | *est.* | *SE* | *p* | *est.* | *SE* | *p* |
| ***PANTING*** | |  |  |  |  |  |
| Intercept | 0.33 | 0.02 | **<0.001** | 3.12 | 0.31 | **<0.001** |
| Window | -0.02 | 0.01 | **0.004** | 0.17 | 0.08 | **0.027** |
| Mass | 0.03 | 0.01 | **0.044** | 0.11 | 0.20 | 0.604 |
| Sex | 0.00 | 0.02 | 0.954 | 0.25 | 0.40 | 0.539 |
| T_a-std_ | -0.11 | 0.02 | **<0.001** | -0.20 | 0.28 | 0.48 |
| ***CALLING*** | |  |  |  |  |  |
| Intercept | 0.29 | 0.03 | **<0.001** | 7.04 | 0.44 | **<0.001** |
| Window | 0.00 | 0.00 | 0.829 | 0.28 | 0.10 | **0.005** |
| Mass | -0.00 | 0.02 | 0.860 | 0.14 | 0.26 | 0.598 |
| Sex | 0.03 | 0.04 | 0.376 | 0.606 | 0.59 | 0.313 |
| T_a-std_ | 0.01 | 0.02 | 0.607 | 1.69 | 0.32 | **<0.001** |

Supplementary table 1. Effects of the onset of panting (n=37 trials for 28 birds) and calling (n=38 trials for 28 birds) on the thermoregulatory variables (LMMs). Window corresponds to the 3 minutes before and after the onset of the behaviour.

|  | | **Fractional mass loss** | | | | |
| --- | --- | --- | --- | --- | --- | --- |
| *Predictors* | | *est.* | | | *SE* | *p* |
| Intercept | | -3.28 | | | 0.07 | **<0.001** |
| Call emission | | 0.16 | | | 0.07 | **0.023** |
| T_a-panting_ | | -0.11 | | | 0.04 | **0.003** |
| Complete | | 0.31 | | | 0.08 | **<0.001** |
| Sex | | 0.08 | | | 0.08 | 0.291 |
| Mass | | 0.02 | | | 0.04 | 0.576 |
|  |  | |  |  | | |

Supplementary table 2. Effects of heat-call emission (1/0) and the T_a_ at the start of panting (T_a-panting_) on the fractional mass loss (LMM, log transformed, n=77 trials for 44 birds).

62 Lighton, J. R. *Measuring metabolic rates: a manual for scientists*. (Oxford University Press, 2008).
